# Anion-exchange chromatography separates structurally heterogeneous and low-potency particles in adeno-associated virus manufacture

**DOI:** 10.1016/j.omta.2026.201812

**Published:** 2026-07-10

**Authors:** Yasuo Tsunaka, Haruka Makihira, Hanano Kono, Zhuolun Yang, Xiaofang Lyu, Sereirath Soth, Mitsuko Fukuhara, Risa Shibuya, Yuki Yamaguchi, Susumu Uchiyama

**Affiliations:** 1 Department of Biotechnology, Graduate School of Engineering, The University of Osaka, 2-1 Yamadaoka, Suita, Osaka 565-0871, Japan; 2 U-Medico Inc., 2-1 Yamadaoka, Suita, Osaka 565-0871, Japan

**Keywords:** adeno-associated virus, AAV, gene therapy, potency, deamidation, EF ratio, quality attribute, manufacturing, AEX, capsid heterogeneity

## Abstract

Recombinant adeno-associated virus (rAAV) manufacturing generates various particles in addition to full particles (FPs), posing a significant challenge for downstream purification. In this study, we applied anion-exchange chromatography (AEX) to resolve such particles of rAAV followed by the characterization of three fractions including an unidentified fraction, termed P3, that elutes at higher salt concentrations. Unexpectedly, density gradient ultracentrifugation (DGUC) clarified that P3 contained both empty particles (P3E) and full particles (P3F). No variation in genome packaging was observed between FPs and P3F, whereas zeta potential measurement showed that P3E and P3F exhibited increased negative surface charge compared with empty particles (EPs) and FPs in the earlier fractions (P1 and P2), respectively. Further characterizations using biolayer interferometry revealed the externalization of the negatively charged N-terminal region in viral protein 1 (VP1) of P3F in P3. In addition, P3F exhibited slightly elevated levels of deamidation, a critical quality attribute associated with vector potency, resulting in their reduced transduction efficiency. Notably, such P3F were not separated by DGUC solely. Collectively, AEX provides an effective strategy for isolating high-quality FPs by removing not only EPs but also structurally altered FPs with reduced potency.

## Graphical abstract

**Figure:**
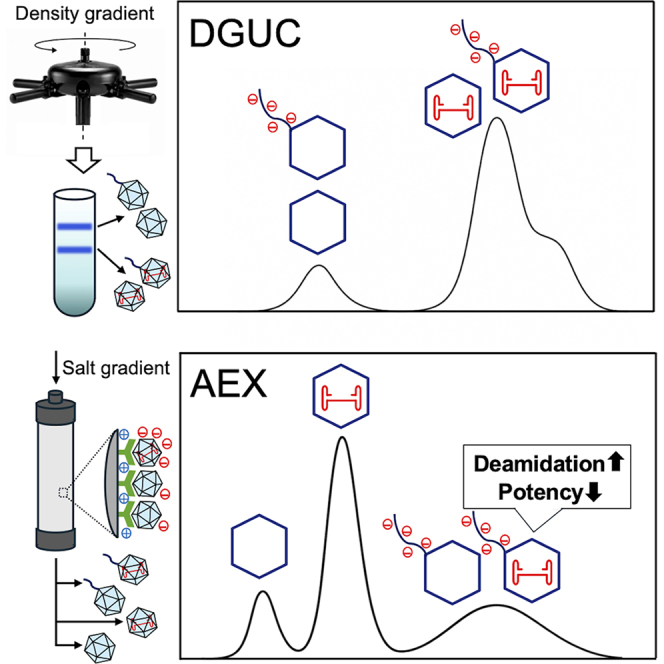

## Teaser

Uchiyama and colleagues demonstrate that anion-exchange chromatography enables separation of structurally heterogeneous adeno-associated virus particles beyond conventional density-based methods. By linking capsid surface charge to externalization of the N terminus of VP1, and deamidation to vector potency, this study provides a conceptual advance for improving product quality in gene therapy manufacturing.

## Introduction

Recombinant adeno-associated virus vectors (rAAVs) have emerged as the leading platform for *in vivo* gene therapy due to their low immunogenicity, broad tissue tropism, and the ability to sustain long-term transgene expression.^1^^,^^2^^,^^3^^,^^4^^,^^5^ The rAAV capsid is a non-enveloped, icosahedral protein shell approximately 25 nm in diameter^6^ and consists of three viral proteins (VPs)—VP1, VP2, and VP3. Although these VPs are encoded by the same cap gene, they differ in molecular weight due to the use of alternative start codons.^7^ Recent studies have shown that VP proteins assemble into 60-mers to form the capsid, but the VP ratio can vary depending on the expression system, batch-to-batch variation in cell culture, and other factors and that differences in the VP ratio can affect biological activity.^8^^,^^9^

VP1 contains the entire VP2 sequence in addition to a unique N-terminal region (VP1u) and possesses phospholipase A2 activity for endosomal escape.^10^ Previous studies suggested that VP1u is normally hidden inside the capsid, but thermal stress can destabilize the capsid and induce VP1u externalization.^11^^,^^12^^,^^13^^,^^14^^,^^15^ Such rAAVs have more negative charge than those with VP1u inside the capsids because VP1u has highly negative charge property. VP2 contains the entire VP3 sequence in addition to a VP1/VP2 common N-terminal region (VP1/2c) and contains at least two nuclear localization signals.^16^ VP stoichiometry, particularly a higher proportion of VP1 and VP2, is positively correlated with rAAV transduction efficiency.^17^^,^^18^

Full particles (FPs) of rAAVs encapsidate a therapeutic single-stranded DNA (ssDNA) transgene.^19^^,^^20^ However, rAAV manufacturing generally yields empty particles (EPs) that lack vector genomes.^21^ EPs are product-related impurities that lack transduction efficiency. These impurities can compromise vector safety and efficacy by increasing immunogenicity and competing for receptor binding, making the removal of EPs a critical requirement for clinical-grade AAV manufacturing.^20^^,^^21^^,^^22^^,^^23^^,^^24^

The typical rAAV manufacturing process involves plasmid transfection into cells, cell lysis, clarification, and affinity chromatography for rAAV capture, followed by a polishing step specifically designed to remove EPs. Historically, density gradient ultracentrifugation (DGUC), which separates EPs and FPs based on their density differences, has been considered the gold standard for this purpose.^25^ However, despite its high-resolution separation capability, DGUC is difficult to scale up and requires open handling, increasing the risk of contamination.^26^ These limitations make DGUC unsuitable for clinical applications.

To address these issues, anion-exchange chromatography (AEX) has gained attention as a robust alternative. The separation principle relies on the subtle difference in surface charge between FPs and EPs.^27^ FPs containing negatively charged DNA exhibit a slightly lower isoelectric point (p*I*) and a stronger net negative charge compared with EPs.^28^ In a typical AEX protocol, AAVs are bound to the column under low ionic strength conditions and eluted by increasing the salt concentration; EPs elute first, followed by the more strongly bound FPs.^29^ On the other hands, AEX faces significant technical challenges, primarily limited resolution. Although various approaches—such as novel column chemistries and Design of Experiments optimization of elution conditions (salt, pH, buffers, additives)—have been explored,^30^^,^^31^^,^^32^^,^^33^^,^^34^ achieving a high EF ratio [EF ratio = FPs/(FPs + EPs)] comparable to DGUC remains difficult.^35^ This has led to the suggestion that optimizing upstream processes to increase the initial EF ratio is also necessary.^36^^,^^37^

Another issue is the incomplete characterization of the elution profile. AEX chromatograms typically display multiple peaks. In particular, three peaks (P1, P2, and P3) were frequently observed.^31^^,^^32^^,^^34^^,^^38^ While P1 and P2 are generally assigned to EP and FP, respectively, the composition of the late-eluting P3 peak remains unclear, despite previous studies describing the physicochemical properties of these fractions.^31^^,^^38^ Understanding these components is essential for process development, product quality assessment, and elucidating the underlying separation mechanisms.

In this study, we isolated individual chromatographic peaks obtained by the CIMmultus QA column and comprehensively characterized their components using multiple analytical approaches, combined with the use of DGUC as a further isolation method. AAV serotypes 8 and 9 carrying an enhanced green fluorescent protein (EGFP) transgene under the control of the cytomegalovirus (CMV) promoter were used as model vectors. We evaluated key quality attributes (QAs), including protein purity, particle size, EF ratio, surface charge, DNA purity, VP stoichiometry, deamidation level, transduction efficiency, and conformation of VP1u. Our analyses revealed several factors influencing AEX separation behavior and demonstrated their associations with vector potency and safety. These findings provide new insights into the mechanisms underlying AEX separation of AAV particles and contribute to improved process development and quality assessment for clinical-grade AAV vectors.

## Results

### AEX chromatographic profiles of AAV8

**Figure 1:**
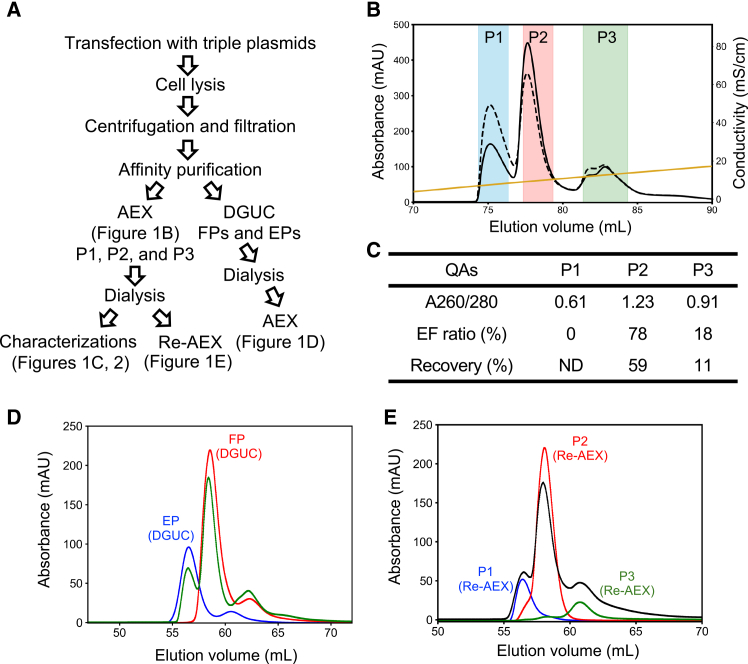
Purification scheme and chromatograms (A) Scheme of the recombinant adeno-associated virus (rAAV) production process in this study. (B) Chromatograms at 280 and 260 nm for anion-exchange chromatography (AEX) purification obtained using a linear sodium chloride gradient. The A280 trace is shown as a dashed line, the A260 trace as a solid line, and the yellow line indicates conductivity. Fractions indicated in blue, red, and green were collected as P1, P2, and P3, respectively. (C) Quality attributes (QAs) of rAAVs in P1, P2, and P3 corresponding to (B). ND, not determined. (D) AEX chromatograms at 260 nm of empty particles (EPs) and full particles (FPs) purified by cesium chloride density gradient ultracentrifugation (DGUC). The elution profile obtained using a linear choline chloride gradient. The blue and red lines represent the elution profiles of EPs and FPs, respectively. The green line represents the AEX chromatogram of the same batch immediately after affinity chromatography. (E) AEX re-chromatograms at 260 nm of P1, P2, and P3 obtained using a linear choline chloride gradient. The blue, red, and green lines represent the elution profiles of P1, P2, and P3 in the second AEX runs, respectively. The black line represents the elution profile from the first AEX run of the same batch.

The AAV8 vectors produced with the triple transfection method using suspended human embryonic kidney 293 (HEK293) cells were purified via the purification processes shown in Figure 1A. Following affinity purification, which exhibited an EF ratio of 25% by mass photometry (MP), AEX purification was performed. The samples were eluted by salt linear gradient (Figure 1B). Three major peaks were observed, consistent with previous studies.^31^^,^^32^^,^^34^^,^^38^ Based on the A260/A280 values, P1 and P2 were expected to originate from EPs and FPs, respectively (Figure 1C). However, based on the A260/A280 value of P3, it was not possible to determine whether this fraction was derived from EPs or FPs. Each peak fraction was collected for subsequent analyses.

**Figure 2:**
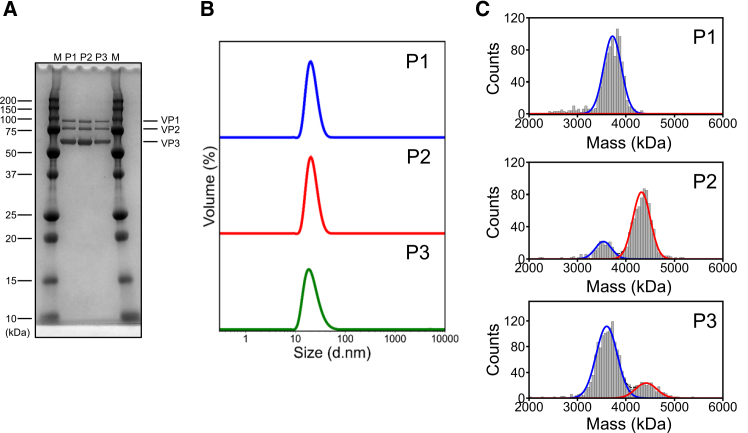
Characterization of P1, P2, and P3 in AEX fractions (A) Protein purity analysis of P1, P2, and P3 fractions by sodium dodecyl sulfate-polyacrylamide gel electrophoresis (SDS–PAGE). M, molecular weight marker. Viral protein 1 (VP1), VP2, and VP3 have theoretical molecular weights of 90, 70, and 62 kDa, respectively. (B) Particle size analysis of P1 (blue), P2 (red), and P3 (green) fractions by dynamic light scattering (DLS). The rAAV monomer size is approximately 25 nm. (C) Full particle ratio (EF ratio) analysis of P1, P2, and P3 fractions by mass photometry (MP). The blue and red lines represent EP and FP populations, respectively.

Sodium dodecyl sulfate-polyacrylamide gel electrophoresis (SDS-PAGE) and dynamic light scattering (DLS) were performed to assess the protein purity of purified rAAV as well as the presence of non-AAV proteins or aggregates. SDS-PAGE showed only bands derived from rAAVs (VP1, VP2, and VP3) (Figure 2A) and DLS showed only AAV monomer peaks (∼25 nm) (Figure 2B), indicating that all the peaks are derived from AAV monomers. As expected, MP analysis showed that P1 contains only EPs and P2 predominantly contained FPs, with 78% EF ratio (Figures 1C and 2C). P3 contained EPs and FPs, with 18% EF ratio (Figures 1C and 2C). Virus genome titers (vg) of the purified rAAVs were quantified via digital polymerase chain reaction (dPCR). Recovery was calculated relative to the input titer before AEX. The recoveries of the P2 and P3 fractions were 59% and 11%, respectively (Figure 1C), suggesting that exclusion of P3 would result in only a limited reduction in the overall yield.

The presence of both FPs and EPs in P3 raises the question of why each particle exhibits an elution peak that is distinct from that of the main peak. To investigate this, we examined the AEX elution profiles of highly enriched FPs and EPs purified by DGUC following affinity chromatography (Figure 1A). Both AEX chromatograms showed two peaks, with the second peaks eluting at the same position as P3 (Figure 1D). These results indicate that P3 represents an overlap of the second peaks derived from both EPs and FPs. Importantly, the second peak of FPs, corresponding to P3, was not resolved by DGUC but was clearly separated by AEX. This highlights the limitation of density-based separation and the advantage of charge-based resolution by AEX.

To confirm the reversibility of the AEX chromatograms of P1, P2, and P3, each fraction from the first run was reloaded onto the AEX column (Figure 1A). Each re-chromatographic peak was eluted at the same conductivity as in the first run (Figure 1E), indicating that the peak components did not undergo interconversion under the conditions. These results suggest that the distinct chromatographic behaviors are relatively stable under the conditions examined.

### Surface charge and DNA impurities in each peak fraction

**Figure 3:**
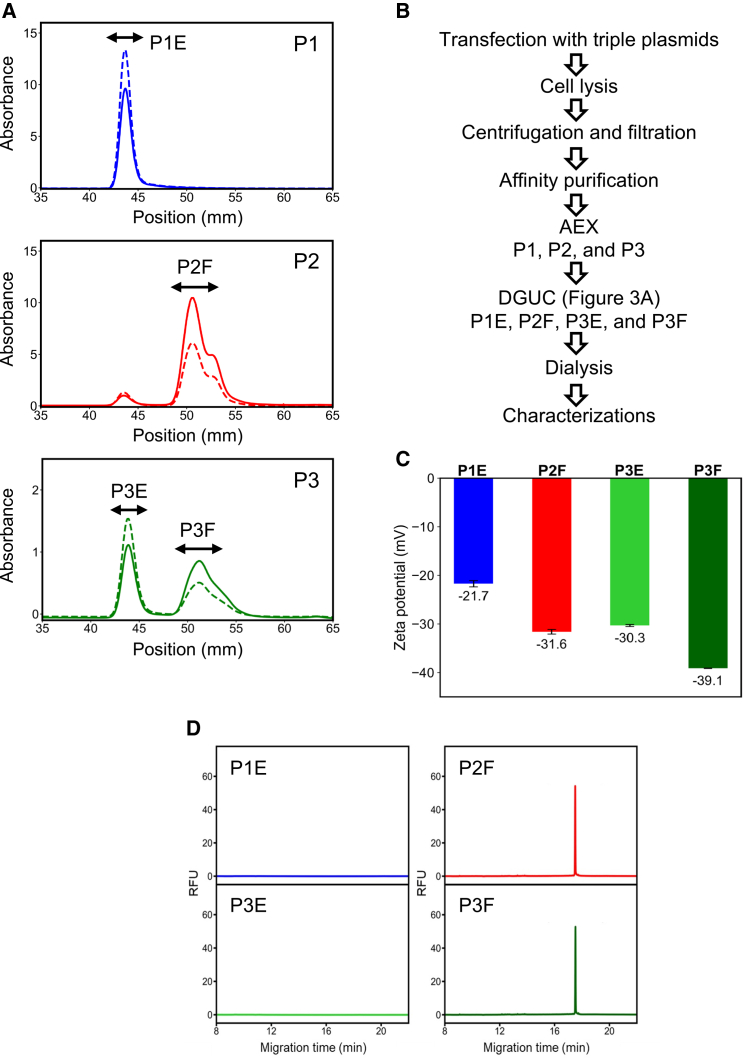
Analysis for surface charge and DNA impurities in each peak fraction (A) DGUC purification of P1 (blue), P2 (red), and P3 (green) fractions. The solid line represents absorbance profiles at 260 nm; the dashed line represents absorbance profiles at 280 nm. Fractions indicated by double-headed arrows were collected. EP fractions from P1 and P3 were collected as P1E and P3E, respectively. FP fractions including their peak tops from P2 and P3 were collected as P2F and P3F, respectively. (B) Scheme of the recombinant adeno-associated virus (rAAV) purification process by DGUC following AEX. (C) Surface charge of P1E (blue), P2F (red), P3E (light green), and P3F (green) fractions by electrophoretic light scattering (ELS). Mean values of triplicate experiments are indicated below each bar. Error bars are ±SD of triplicate experiments. (D) DNA purity analysis of P1E (blue), P2F (red), P3E (light green), and P3F (green) fractions by capillary gel electrophoresis-laser-induced fluorescence (CGE-LIF). A single peak was detected in the FP fractions.

To clarify the differences among the components of each peak, we need to perform further characterization. However, P3 contains both EPs and FPs, which were expected to possess distinct properties. To separately analyze EPs and FPs, each peak was separated to EPs and FPs by the DGUC purification following AEX (Figures 3A and 3B). EPs from P1 and P3 were collected as P1E and P3E, respectively. FPs from P2 and P3 were collected as P2F and P3F, respectively.

To estimate their surface charges, zeta potentials were measured by electrophoretic light scattering (ELS). We first evaluated zeta potentials in the AEX equilibration buffer containing 10 mM MgCl_2_. Under these conditions, P2 and P3 exhibited zeta potentials of approximately −0.7 mV and −1.4 mV, respectively, indicating the same trend observed in the AEX elution profiles. However, the magnitude of the measured zeta potentials was small, making it difficult to clearly discriminate between the fractions. MgCl_2_ was, therefore, excluded from subsequent measurements because electrostatic shielding by Mg^2+^ can suppress the measured zeta potential. Accordingly, the samples were dialyzed against the AEX equilibration buffer lacking MgCl_2_. As shown in Figure 3C, P3E and P3F were more negatively charged than P1E and P2F, respectively, broadly consistent with the AEX elution profiles. However, the similar values of P2F and P3E did not correlate with their elution behavior in AEX. This may be due to differences in buffer conditions in the absence of MgCl_2_. Magnesium ions are known to significantly impact AEX elution behavior, as previously reported.^39^ In particular, the inclusion of MgCl_2_ has been shown to improve the resolution between EPs and FPs. Consistent with this notion, when AEX was performed in the absence of MgCl_2_, P3 was no longer clearly separated and largely overlapped with P2 (Figure S1), indicating that the charge differences measured by zeta potential are associated with the observed chromatographic behavior.

The underlying cause of the charge heterogeneity in P3 could be DNA impurity contained in the rAAV capsid. To investigate the possibility, DNA impurity in each fraction was analyzed by capillary gel electrophoresis-laser-induced fluorescence (CGE-LIF). However, only the main peak of ssDNA was detected in the P2F and P3F fractions, and no DNA impurities were detected in any fraction (Figure 3D).

### Capsid heterogeneity in each peak fraction

**Figure 4:**
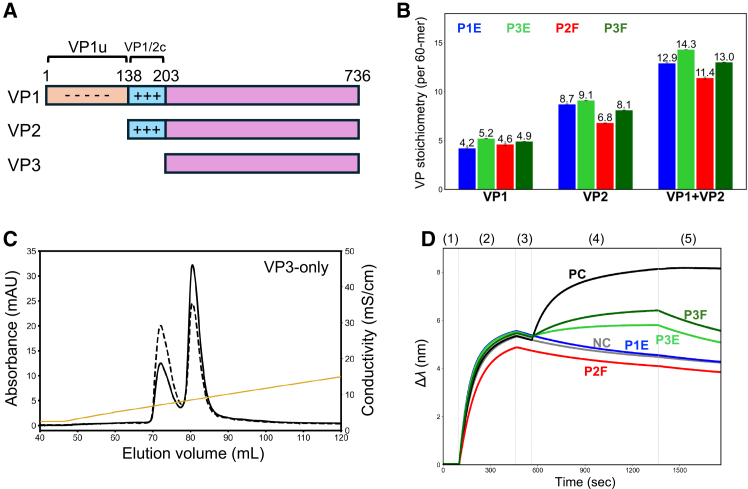
Analysis of capsid heterogeneity in each peak fraction (A) Schematic representation of the VP1, VP2, and VP3 domain structures and their charge properties. The negatively charged VP1-unique region (VP1u) is present only in VP1, whereas the positively charged VP1/2 common region (VP1/2c) is shared between VP1 and VP2. VP3 consists solely of the common capsid region. Numbers indicate amino acid positions. Plus (+) and minus (−) symbols indicate relatively positive and negative charge distributions, respectively. (B) VP stoichiometry of P1E (blue bars), P2F (red bars), P3E (light green bars), and P3F (green bars) fractions by capillary gel electrophoresis-sodium dodecyl sulfate (CGE-SDS). VP stoichiometry (per 60-mer) was calculated based on the peak area ratio in CGE-SDS electropherogram and molar extinction coefficients of VP1, VP2, and VP3. Mean values of triplicate experiments are indicated above each bar. Error bars represent ±SD of triplicate experiments. (C) AEX chromatogram at 280 and 260 nm of VP3-only, which is an AAV mutant with a capsid consisting entirely of VP3. The elution profile obtained using a linear sodium chloride gradient. The A280 trace is shown as a dashed line, the A260 trace as a solid line, and the yellow line indicates conductivity. (D) Binding analysis of rAAVs by biolayer interferometry (BLI). A1 antibody-immobilized condition following five steps: (1) baseline measurement, (2) loading of A1 antibody, (3) baseline measurement, (4) association with rAAVs, and (5) dissociation from AAVs, which are separated by dotted lines in the figure. The blue, red, light green, and green lines represent the P1E, P2F, P3E, and P3F fractions introduced during the association step (4), respectively. The positive control (PC) is a heated FP sample (black line), and the negative control (NC) is the AEX equilibration buffer (gray line).

Because each VP has a different p*I* due to the differences in their N-terminal regions (Figure 4A),^28^ the observed charge heterogeneity may arise from the variations in VP stoichiometry within the rAAV capsid. To investigate this possibility, VP stoichiometry was analyzed by capillary gel electrophoresis-sodium dodecyl sulfate (CGE-SDS). As shown in Figure 4B, the proportion of VP1, which contains both the negatively charged VP1u region and the positively charged VP1/2c region (Figure 4A), was slightly higher in P3 than in P1 and P2. However, the proportion of VP2, which contains the positively charged VP1/2c region (Figure 4A), was also increased in P3. As a result, although P3 exhibited higher VP1 and VP2 stoichiometry, these differences could not fully explain the charge variations among the peaks, suggesting contributions from factors other than VP stoichiometry.

To investigate whether the charge-rich VP1u and VP1/2c regions contribute to the charge heterogeneity of P3, the affinity-purified VP3-only, which is an AAV mutant with a capsid consisting entirely of VP3,^14^^,^^40^ was prepared, and the AEX elution behavior was compared with that of the wild type (Figure 1B). Surprisingly, VP3-only did not show P3 in AEX (Figure 4C), indicating that the separation behavior of P3 in AEX is attributed to the presence or absence of the VP1u and/or VP1/2c regions. While VP3-only capsids may not fully recapitulate the physicochemical properties of wild-type particles, a previous study has demonstrated that they form structurally intact capsids.^41^ Consistent with this report, MP analysis of the VP3-only preparation confirmed the presence of assembled capsids with a particle mass distribution and EF ratio comparable to those of the wild-type preparation (Figures 2C and S2). In addition, VP3-only FPs have been reported to exhibit thermal stability comparable to that of wild-type FPs.^14^ Consistent with this report, our previous forced degradation study showed that VP3-only FPs exhibited a decrease in particle mass comparable to that observed for wild-type FPs.^15^ Taken together, these findings suggest that VP3-only capsids do not possess markedly different assembly properties or overall stability from wild-type capsids. These observations support the interpretation that the absence of P3 is not attributable to altered capsid assembly or stability in the VP3-only preparation.

In a previous study, VP1u was shown to be typically hidden inside the capsid, whereas thermal stress can destabilize the capsid and induce the externalization of VP1u on the capsid surface.^14^ Such AAVs may exhibit a higher negative charge on the capsid surface than AAVs with VP1u retained inside the capsid, as VP1u carries a strong negative charge (p*I* < 5).^28^ Accordingly, the increased negative charge of P3 AAVs is likely due to the externalization of the negatively charged VP1u. To test this hypothesis, the binding signal of the A1 antibody, which specifically binds to VP1u, was detected using biolayer interferometry (BLI). As shown in Figure 4D, the binding response to the A1 antibody was detected in the heated FP sample, whereas P1E and P2F did not show the binding. In contrast, both EPs and FPs in P3 (P3E and P3F) showed the binding response to A1 antibody, indicating rAAVs in P3 possess the properties of their VP1u externalized on the capsid surface. A control experiment without A1 antibody immobilization confirmed that the binding signal was not due to non-specific sensor binding.

### Deamidation and transduction efficiency in each peak fraction

**Figure 5:**
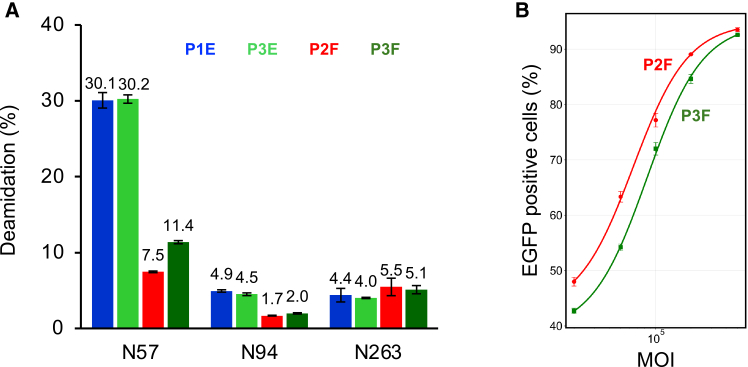
Analysis of deamidation and transduction efficiency in each peak fraction (A) Deamidation levels of P1E (blue bars), P2F (red bars), P3E (light green bars), and P3F (green bars) fractions analyzed by peptide mapping. Asparagine residues with deamidation levels exceeding 1% are shown. Mean values of triplicate experiments are indicated above each bar. Error bars are ±SD of triplicate experiments. (B) *In vitro* transduction efficiency comparing P2F (red) and P3F (green) fractions. Cells were transduced at the indicated multiplicities of infection (MOIs; vector genomes per cell) of 2 × 10^4^, 5 × 10^4^, 1 × 10^5^, 2 × 10^5^, and 5 × 10^5^. Error bars are ±SD of the mean of triplicate experiments. Dose-response curves were fitted using a sigmoidal regression model and compared using an *F*-test. A significant difference was observed between P2F and P3F (*p* = 0.0429).

Deamidation increases the net negative charge of the AAV capsid, leading to prolonged retention in analytical AEX for AAV8,^15^ and is considered a potential critical QA related to vector potency and safety. In fact, deamidation of some asparagine residues has been reported to decrease the transduction efficiency of rAAV.^42^^,^^43^ Deamidation levels of FPs and EPs in each peak fraction were examined by peptide mapping using liquid chromatography (LC)-tandem mass spectrometry (MS). Sequence coverages for all samples were >93%, covering 56 of 58 asparagine residues that could potentially undergo deamidation. Among the identified sites, residues N57, N94, and N263 showed deamidation levels >1%. Between P2F and P3F, the difference in deamidation level was <0.5% at all residues except N57, which exhibited an approximately 4% higher deamidation level in P3F (Figure 5A). In addition, the differences in deamidation levels between P1E and P3E were <0.5% at all residues. These results suggest that deamidation alone is unlikely to fully account for the increased AEX retention observed in P3.

HeLaRC32 cells were transduced with DGUC-purified FPs from P2 and P3 (P2F and P3F) at various multiplicities of infection (MOIs), and transduction efficiencies were evaluated based on the percentage of EGFP-positive cells (Figure 5B). Consistent with the differences in deamidation levels, the transduction efficiency of P3F was significantly lower than that of P2F. Sigmoidal regression analysis demonstrated a significant difference between P2F and P3F (*F*-test, *p* = 0.0429; Figure 5B).

### Effects of forced stress on P2 fraction

**Figure 6:**
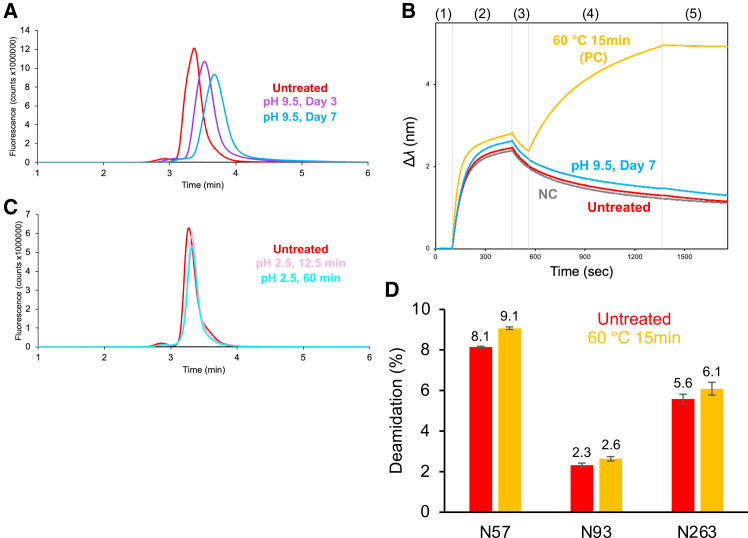
Analysis of effects of forced stress on P2 fraction (A) Analytical AEX chromatograms of alkaline-treated P2 samples stored at 25°C and pH 9.5 for 0 (red), 3 (purple), and 7 (light blue) days. Fluorescence detection is shown. (B) Binding analysis of alkaline-treated P2 samples by BLI. Experimental conditions were the same as those described in Figure 4D. The red and light blue lines represent the P2 samples stored for 0 and 7 days introduced during the association step (4), respectively. The positive control (PC) is an AAV8 P2 sample heated at 60°C for 15 min (orange line), and the negative control (NC) is the formulation buffer (gray line). (C) Analytical AEX chromatograms of acidic-treated P2 samples stored at 25°C and pH 2.5 for 0 (red), 12.5 (pink), and 60 (cyan) mins. Fluorescence detection is shown. (D) Deamidation levels of untreated P2 (red bars) and P2 heated at 60°C for 15 min (orange bars) determined by peptide mapping. Three deamidation-prone asparagine residues (N57, N93, and N263) are shown. Values represent the mean of triplicate experiments, with error bars indicating ±SD.

To further investigate the contribution of deamidation to AEX retention and VP1u externalization, P2 particles of AAV8 were subjected to alkaline treatment, which is known to promote deamidation at N57, N66, N94, and N263,^15^ followed by analytical AEX and BLI. Alkaline treatment at pH 9.5 and 25°C for 0, 3, and 7 days resulted in a progressive shift of the elution profile toward higher salt concentrations (Figure 6A), indicating that deamidation contributes to increased retention on the AEX column. BLI analysis was performed on samples treated for 0 and 7 days (Figure 6B). No binding response to the A1 antibody was detected in either sample, indicating that deamidation alone is insufficient to induce detectable VP1u externalization.

To evaluate whether the transient acidic exposure encountered during affinity chromatography affects AEX retention, the P2 particles were incubated at pH 2.5 and 25°C for 0, 12.5, and 60 min, mimicking the acidic exposure during affinity purification, followed by analytical AEX. Analytical AEX showed no appreciable shift in the elution profile of the sample with the acidic treatment (Figure 6C). These results indicate that transient acidic exposure has little effect on AEX retention under the conditions examined.

To determine whether heat-induced VP1u externalization is accompanied by deamidation, peptide mapping of the P2 particles was performed before and after the incubation at 60°C for 15 min. The heat treatment resulted in only slight increases in deamidation, with increases of less than 1% at the three deamidation sites analyzed (Figure 6D). Despite these slight increases in deamidation, the heat-treated P2 fraction, used as a positive control, exhibited detectable VP1u externalization by BLI (Figure 6B).

### AEX chromatographic profiles and BLI analysis of AAV9

**Figure 7:**
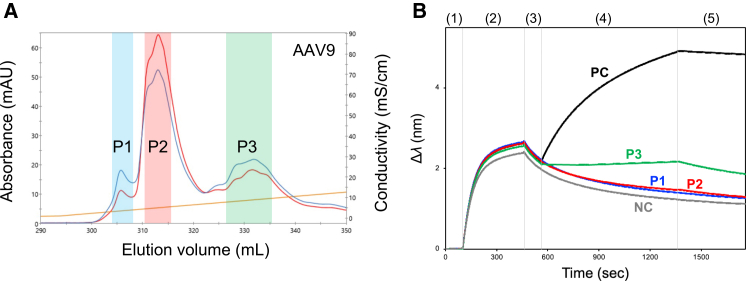
AEX chromatographic profiles and BLI analysis of AAV9 (A) Chromatograms at 280 and 260 nm for AEX purification of affinity-purified AAV9 using a linear sodium chloride gradient. The A280 trace is shown as a blue line, the A260 trace as a red line, and the yellow line indicates conductivity. Fractions indicated in blue, red, and green were collected as P1, P2, and P3, respectively. (B) Binding analysis of AAV9 by BLI. Experimental conditions were the same as those described in Figure 4D. The blue, red, and green lines represent the P1, P2, and P3 fractions introduced during the association step (4), respectively. The positive control (PC) is a heated AAV9 P2 sample (black line), and the negative control (NC) is the formulation buffer (gray line).

To assess whether the observed chromatographic behavior and the externalization of VP1u is unique to AAV8, AAV9 vectors produced by triple transfection of suspension HEK293 cells were purified by affinity chromatography, and subsequently subjected to AEX purification. As observed for AAV8 (Figure 1B), the AEX chromatogram of AAV9 also contained a distinct P3 population (Figure 7A). This observation suggests that the chromatographic heterogeneity is not unique to AAV8 and may be relevant to other AAV serotypes.

BLI analysis was performed on the P1, P2, and P3 fractions of AAV9 separated by AEX (Figure 7A). The binding response to the A1 antibody was also detected in the P3 fraction of AAV9, whereas P1 and P2 did not show the binding (Figure 7B), indicating that AAV9 in P3 also possesses the properties of the VP1u externalization on the capsid surface. These findings suggest that the VP1u externalization of P3 is not unique to AAV8.

## Discussion

In rAAV manufacturing, various process- and product-related impurities are co-produced alongside FPs. A major challenge is the separation of EPs, FPs, partial particles, and structurally heterogeneous particles. Our study revealed that the high-salt elution peak P3 in AEX (Figure 1B) consists of EPs and FPs with externalized VP1u on the capsid surface (Figure 4D) and demonstrates that AEX can separate such particles. Given that only the main ssDNA peak was detected in FPs in P2 and P3 and no DNA impurities were observed (Figure 3D), the heterogeneity represented by P3 is unlikely to arise from genome packaging, but rather from variations in capsid surface structure. Characterization regarding the safety and efficacy of these AAV particles showed that FPs in P3 had higher deamidation level at the N57 residue and reduced transduction efficiency compared with FPs in P2 (Figure 5). It has been reported that deamidation at residue N57 negatively correlates with the transduction efficiency,^18^ which is consistent with the lower transduction efficiency of FPs in P3 (Figure 5B). Therefore, AEX may be a useful method for selectively enriching high-quality FPs. Taken together, these results suggest that P3 should be excluded during AEX purification, as it contains EPs as well as structurally altered, lower-quality FPs.

Importantly, such structurally altered FPs were not removed by DGUC (Figures 1B and 1D), as this method separates particles based on buoyant density rather than surface properties. In contrast, AEX effectively resolves these variants based on charge differences, enabling the isolation of a more homogeneous FP population. These findings highlight the advantage of AEX for AAV purification, as it allows the removal of structurally heterogeneous and potentially lower-potency particles that are indistinguishable by conventional density-based methods.

Previous studies have shown that stress conditions induce changes in AEX profiles of rAAV, which have been attributed to capsid destabilization, resulting in charge alterations through deamidation and the externalization of the N-terminal regions of VP.^14^^,^^15^^,^^44^ These findings indicate that structural changes in capsid proteins can directly impact surface charge and chromatographic behavior. In line with these observations, our results suggest that the P3 fraction represents structurally altered particles characterized by increased AEX retention (Figure 1B). To clarify the contribution of deamidation to the increased AEX retention of P3, P2 particles were subjected to an alkaline treatment, which has previously been shown to increase deamidation at multiple asparagine residues (N57, N66, N94, and N263).^15^ The alkaline treatment of P2 induced a progressive shift toward the P3-like chromatographic profile (Figure 6A). These findings indicate that deamidation contributes to the increased AEX retention. However, the alkaline treatment did not induce detectable VP1u externalization (Figure 6B), indicating that deamidation alone is insufficient to trigger VP1u externalization. In fact, in the P3 fraction generated during AAV manufacturing, the deamidation level increased by only approximately 4% at N57 in FPs compared with P2 and was not elevated in EPs compared with P1 (Figure 5A). Our previous studies demonstrated that VP1u externalization is associated with heat-induced genome release.^14^ In this study, the heat treatment induced detectable VP1u externalization (Figure 6B) despite only slight increases (<1%) in deamidation at the three analyzed sites (Figure 6D), indicating that extensive deamidation is not required for VP1u externalization. These findings suggest that capsid destabilization, including that induced by thermal stress, may facilitate conformational changes that promote VP1u externalization. Together, these findings indicate that both deamidation and VP1u externalization contribute to the increased AEX retention of P3 during AAV manufacturing, whereas, given the modest increase in deamidation observed in P3 FPs (approximately 4% at N57), VP1u externalization is likely to be a major determinant.

To better understand the cause of P3 emergence during rAAV manufacturing, we evaluated the effects of representative process-related stress conditions on AEX behavior through deamidation and/or VP1u externalization of rAAV. The alkaline treatment increased AEX retention (Figure 6A), whereas transient acidic treatment, which mimics the acidic exposure during affinity chromatography, had little effect on chromatographic behavior (Figure 6C). As for the control sample, which was generated by the heat treatment, detectable VP1u externalization was observed (Figure 6B) despite marginal increases in deamidation (Figure 6D). Collectively, these findings suggest that transient acidic exposure during affinity purification is unlikely to be a major driver of P3 formation, whereas conditions that promote deamidation or induce capsid destabilization are more likely to contribute to P3 generation. These results imply that process conditions capable of promoting deamidation or inducing capsid destabilization may increase the risk of generating structurally altered particles during rAAV manufacturing. Taken together, these findings indicate that AEX may serve not only as a purification step but also as a sensitive analytical tool for monitoring stress-induced capsid heterogeneity generated during rAAV manufacturing.

A previous study has reported that AEX-separated AAV fractions exhibit differences in VP composition, with the later-eluting fractions showing altered VP1/VP2/VP3 ratios and increased levels of capsid protein variants.^38^ These findings suggest that variations in VP stoichiometry can contribute to changes in capsid surface charge and chromatographic behavior. In addition, it has been suggested that particles with stronger AEX retention tend to exhibit increased negative charge associated with capsid protein modifications and compositional differences. VP stoichiometry analysis demonstrated that P3 AAVs contain slightly higher proportions of VP1 and VP2 compared with P1 and P2 (Figure 4B). In a previous study, the VP1u region was shown to be predominantly located inside the capsid.^11^ Therefore, an increased proportion of VP1 and VP2 may lead to greater internal crowding and structural constrain within the capsid, potentially compromising capsid stability.^45^ Such destabilization may facilitate the externalization of the VP1u region, resulting in increased exposure of negatively charged domains on the capsid surface. This mechanism provides a plausible explanation for the enhanced negative charge and distinct chromatographic behavior observed for P3 AAVs. This interpretation is consistent with the notion that AEX can resolve capsid heterogeneity arising from differences in VP composition and structural alterations.

In conclusion, our study demonstrates that the P3 fraction arises from structural variations in AAV capsids rather than differences in genome packaging. The increased externalization of the negatively charged VP1u region is considered to contribute to the enhanced surface charge observed in P3. Such structurally heterogeneous particles could not be resolved by conventional density-based methods such as DGUC, highlighting a key limitation of these approaches. In contrast, AEX enables the separation of AAV particles based on subtle differences in capsid surface properties, allowing the isolation of higher-purity FPs. These findings underscore the importance of charge-based separation strategies for improving rAAV product quality and provide a mechanistic basis for the selective removal of structurally altered, low-potency particles during purification. Importantly, we found that AAV9, in addition to AAV8, also exhibited a distinct P3 population with VP1u externalization (Figure 7), suggesting that this chromatographic heterogeneity is not unique to a single serotype. These findings indicate that AEX-based separation of structurally altered particles may be broadly applicable across multiple AAV serotypes. In addition, even in cases where rAAV manufacturing unexpectedly yields particles exhibiting capsid structural alterations, including VP1u externalization and deamidation, AEX can potentially separate and reduce the population of these variants based on charge differences, providing a robust strategy to control product quality. Together, this work highlights AEX as a powerful platform not only for purification but also for resolving hidden capsid heterogeneity that directly impacts vector quality.

## Materials and methods

### Cell culture

As the suspended HEK293 cell line, Viral Production Cells 2.0 (VPC2.0; Thermo Fisher Scientific, Waltham, MA) were grown in BalanCD HEK293 medium (FUJIFILM Irvine Scientific, Santa Ana, CA) with 6 mM L-glutamine (FUJIFILM Wako Pure Chemical Industries, Tokyo, Japan) and used for AAV vector production.

### rAAV production

The transgene plasmid (CMV-EGFP), pRep2Cap8 for AAV8 or pRep2Cap9 for AAV9, and pHelper were co-transfected into suspended VPC2.0 cells (2.0 × 10^6^ cells/mL) at a plasmid ratio of 1:1:1, and the cells were cultured in flasks. FectoVIR-AAV (Sartorius Polyplus, Illkirch, France) was used as the transfection reagent. At approximately 72–96 h post-transfection, the medium and cell lysates were harvested. The lysates were clarified by centrifugation at 4,000 × *g* for 20 min and filtration through a 0.22-μm polyether sulfone filter (Sartorius, Goettingen, Germany).

### Affinity purification

The clarified lysates were loaded onto POROS GoPure AAVX prepacked columns (Thermo Fisher Scientific) equilibrated with 0.15 M NaCl, 20 mM Tris (pH 7.6), and 0.001% (w/v) poloxamer 188 (P188; BASF, Ludwigshafen, Germany) using an ÄKTA system (Cytiva). After washing with 1 M NaCl, 20 mM Tris (pH 7.6), and 0.001% P188, the AAV was eluted by low-pH elution in 0.1 M glycine (pH 2.5), 0.4 M L-arginine, and 0.001% P188. The eluate was collected, pooled, and neutralized.

### AEX purification

AEX for AAV8 was performed using an ÄKTA system (Cytiva) equipped with a 1 mL CIMmultus QA monolith column (Sartorius BIA Separations). The column was equilibrated with 20 mM Tris-HCl (pH 9.0), 1% sucrose, 10 mM MgCl_2_, and 0.001% P188. The affinity-purified samples were diluted with the equilibration buffer lacking MgCl_2_ and loaded onto the column. After washing with the equilibration buffer, AAV vectors were eluted using a linear gradient of NaCl or choline chloride. Most AEX experiments were performed using a linear NaCl gradient, whereas choline chloride was used only in some experiments, as indicated in the figure legends. Comparable chromatographic profiles were obtained using either NaCl or choline chloride as the eluting salt (Figure S3), suggesting that the observed peak heterogeneity was not dependent on the choice of the counterion. AEX for AAV9 was performed with a 1-mL CIMmultus QA HR monolith column (Sartorius BIA Separations). The column was equilibrated with 20 mM Tris-HCl (pH 9.0), 2 mM MgCl_2_, and 0.001% P188. The AC-purified samples were diluted with the equilibration buffer and loaded onto the column. After washing with the equilibration buffer, AAV vectors were eluted using a linear gradient of NaCl. Fractions corresponding to each peak (P1, P2, and P3) were collected separately, and in selected lots, these fractions were further purified by DGUC, as described below. Finally, the AEX-purified samples were dialyzed into formulation buffer (200 mM NaCl, 1× phosphate-buffered saline [PBS], and 0.001% P188) by Slide-A-Lyzer 10K (Thermo Fisher Scientific).

### DGUC purification

The affinity-purified samples were also subjected to cesium chloride DGUC using Optima XE-90 Ultracentrifuge (Beckman Coulter) to enable clear separation of the FPs and EPs. DGUC conditions are as follows; 3.5 M CsCl with 1× PBS and 0.001% P188, 18,000 rpm, 16°C, 24 h. Finally, the collected FP and EP samples were dialyzed into formulation buffer (200 mM NaCl, 1× PBS, and 0.001% P188) by Slide-A-Lyzer 10K (Thermo Fisher Scientific).

For the AEX-purified samples, DGUC was performed to separate the EPs and FPs in each fraction, completely. Each peak fraction was transferred to an ultracentrifugation tube containing 2.5 M CsCl in 1× PBS with 0.001 w/v% poloxamer 188. The samples were then centrifuged at 24,000 rpm using an Optima XE-90 ultracentrifuge (Beckman Coulter) equipped with a SW41Ti rotor at 16°C for 72 h. Fractions corresponding to EPs from P1 and P3 were collected as P1E and P3E, respectively. FP fractions from P2 and P3 were isolated and pooled as P2F and P3F. A portion of them was dialyzed against the AEX equilibration buffer for ELS analysis, while the remainder was dialyzed against the formulation buffer (200 mM NaCl, 1× PBS, and 0.001% P188) by Slide-A-Lyzer 10K (Thermo Fisher Scientific).

### dPCR

Purified rAAVs were diluted in dilution buffer, which consisted of DNase I buffer, DNase I (Takara Bio, Shiga, Japan), and 0.05 w/v% poloxamer 188 (BASF, Ludwigshafen, Germany), and incubated at 37°C for 30 min. DNase I was then inactivated by adding EDTA (pH 8.0; NIPPON GENE, Tokyo, Japan) to a final concentration of 50 mM and incubated at 25°C for 5 min and at 95°C for 10 min. The pretreated samples were diluted with 0.001 w/v% poloxamer 188 in TE (NIPPON GENE, Tokyo, Japan) to the required concentration. dPCR was performed as a duplex assay using specific forward and reverse primers (900 nM) and a specific probe (250 nM) that targeted the EGFP using a fluorescein amidite fluorophore (Hokkaido System Science, Hokkaido, Japan). Reaction mixes comprised primers, probes, nuclease-free water, and QuantStudio Absolute Q DNA Master Mix (Thermo Fisher Scientific). The dPCR was performed using a QuantStudio Absolute Q Digital PCR System and 16-well microfluidic array plates (Thermo Fisher Scientific). The dPCR sequence consisted of enzyme activation at 94°C for 10 min, followed by 40 cycles of 94°C for 5 s and 54°C for 30 s. The following primers and probes were used: EGFP forward primer 5′-GGA GCG CAC CAT CTT CA-3′, EGFP reverse primer 5′-CAG GGT GTC GCC CTC GA-3, and EGFP probe 5′-6FAM- CTA CAA GAC CCG CGC CGA GGTG-MGB-3′.

### SDS-PAGE

Each sample was mixed with 6× sample buffer solution with reducing reagent for SDS-PAGE (Nacalai Tesque); for lanes P1 and P2, 10 μL of the sample was mixed with 2 μL of the sample buffer, and for lane P3, 15 μL of the sample was mixed with 3 μL of the sample buffer. The mixtures were incubated at 95°C for 3 min and subjected to SDS-PAGE using a precast 15% polyacrylamide gel in 1× SDS-containing running buffer, diluted from a 10× running buffer solution for SDS-PAGE (Nacalai Tesque). A prestained protein marker (Precision Plus Protein All Blue Prestained Protein Standards, Bio-Rad; 10 μL per lane) and samples (P1 and P2: 12 μL; P3: 18 μL) were loaded, and electrophoresis was performed at a constant current of 30 mA for 1 h. After electrophoresis, the gel was washed twice with Milli-Q water for 3 min each, stained with Coomassie brilliant blue staining solution for 20 min with gentle agitation, and destained with Milli-Q water until background staining was sufficiently reduced. Protein bands were visualized using an iBright CL1500 Imaging System (Thermo Fisher Scientific).

### DLS

DLS measurements were performed using Zetasizer Ultra (Malvern Panalytical Ltd., Malvern, UK). DLS measurements were performed by placing 30 μL in a low-volume quartz batch cuvette (ZEN2112). The temperature at the time of measurement was 25°C. Each sample was measured in triplicate.

### MP

MP measurements were performed using a TwoMP instrument (Refeyn). Coverslips (24 × 50-mm precision; Thorlabs, Newton, NJ) were prepared by cleaning with Milli-Q water and ethanol. A piece of precut 2 × 3 culture well gasket (Grace Bio-Labs, Bend, OR) was placed onto the coverslip. For MP measurements, 16–19 μL of 1× PBS was loaded into the well created by the gasket, and the focus was automatically adjusted. Then, 1–4 μL of the sample was added to the same wells to produce a final volume of 20 μL, and the samples were mixed by pipetting. Next, 60 s of movie data were recorded. Mass calibration was performed using apoferritin (Sigma-Aldrich) and VP3-only EP. The histograms of the mass distributions were peak fitted with a Gaussian function for EPs and FPs using the in-house Python script.

### ELS

ELS measurements were recorded on a Zetasizer Ultra instrument (Malvern Panalytical Ltd., Malvern). Measurements were performed using the diffusion barrier method in which 20- to 120-μL aliquots of the samples were introduced into a folded capillary cell (DTS1070 Malvern Panalytical Ltd., Malvern) containing the AEX equilibration buffer lacking MgCl_2_. The instrument settings were optimized automatically by means of the ZS XPLORER software. All measurements were made at 25°C and consisted of (1) three repeat DLS size measurements using backscatter detection, (2) five repeat ELS zeta potential measurements, and (3) three repeat DLS size measurements using backscatter detection. The use of DLS size measurements before and after the ELS measurements was to confirm that the application of a voltage did not cause the integrity of the samples to be compromised. The measured electrophoretic mobilities were converted into zeta potentials using the Smoluchowski approximation.

### CGE-LIF

First, 20 mg/mL proteinase K (QIAGEN, Hilden, Germany) was added to each sample (1.0 × 10^11^ viral capsids) and incubated at 55°C for 60 min. Subsequently, the sample was incubated at 95°C for 20 min to digest the capsid, and the lysate was collected by centrifugation. The collected lysate was purified, and electrophoresis was performed in a capillary filled with 5 mL of Nucleic Acid Extended Range Gel (SCIEX, Framingham, MA) containing 10 μL of SYBR GreenII RNA Gel Stain (SCIEX). DNA was measured using a PA 800 Plus (SCIEX) instrument with 488-nm laser excitation fluorescence.

### CGE-SDS

rAAV samples (5.0 × 10^10^ viral capsids) were denatured with 18 mg/mL SDS and 5.98% 2-mercaptoethanol and buffer exchanged twice for desalting with Amicon Ultra-0.5 Ultracell-30 kDa (Merck, Darmstadt, Germany) and Matrix Exchange Solution (0.33 mg/mL SDS and 3.5% 2-mercaptoethanol). The final collected samples were diluted with 50 μL of deionized water for injection. CGE-SDS measurements were performed using the PA800 plus system (SCIEX). The prepared samples were injected with water plug sample stacking. Detection was performed at 214 nm using a photodiode array detector. Molar extinction coefficients (M^−1^ cm^−1^) of VP1, VP2, and VP3 used for VP stoichiometry calculations were 1,729,736, 1,421,965, and 1,317,147, respectively.^46^

### Peptide mapping

Peptide mapping method was performed as described previously,^15^ with minor modifications. 7.5 μg of each rAAV sample was freeze-dried for 2 h and dissolved in 6 M guanidine hydrochloride, 5 mM tris(2-carboxyethyl) phosphine hydrochloride (FUJIFILM Wako Pure Chemical), 25 mM iodoacetamide (FUJIFILM Wako Pure Chemical), and 50 mM ammonium bicarbonate (FUJIFILM Wako Pure Chemical). To the denatured rAAV, we added 400 μL of cold methanol (Nacalai Tesque), 100 μL of chloroform (Kanto Chemical, Tokyo, Japan), and 300 μL of water; then we vortexed the mixture and centrifuged it at 4°C and 18,000 × *g* for 10 min. Subsequently, the upper layer was removed, 300 μL of cold methanol was added, and the mixture was vortexed and centrifuged at 4°C and 19,000 × *g* for 10 min. Afterward, the supernatant was removed, and the tube was dried for 5 min. RapiZyme trypsin (Waters, Milford, MA) and lysyl endopeptidase R (Lys-C; FUJIFILM Wako Pure Chemical) were added at a trypsin:Lys-C:rAAV ratio of 2:1:15 in 50 mM ammonium acetate, pH 6.5, followed by incubation at 37°C for 2 h. The reaction was quenched with trifluoroacetic acid to a final concentration of 0.1%. Peptides were analyzed using an Ultimate 3000 RSLC nano LC system coupled to a Q Exactive HFX mass spectrometer (Thermo Fisher Scientific). The peptides were separated using an Acquity UPLC Peptide CSH C18 Column (130 Å, 1.0 × 150 mm; 1.7 μm particle size) at 45°C. Mobile phase A consisted of water containing 0.1 v/v % formic acid (FA), and mobile phase B consisted of acetonitrile containing 0.1 v/v % FA. Separation was performed using a gradient of 5%–35% mobile phase B over 45 min at 50 μL/min. A full-MS scan was performed using an ion transfer tube temperature of 250°C, spray voltage of 3.5 kV, a resolution of 120,000, a mass range (m/z) of 300–2,000, and a funnel radio frequency level of 40. A subsequent data-dependent tandem MS scan was performed using a higher-energy collisional dissociation of 27% and a resolution of 30,000. The peptides were identified, and the deamidation rates were quantified using a Byos v.5.12.25 (Protein Metrics, Boston, MA).

### *In vitro* transduction efficiency assay

HeLaRC32 expresses the Rep and Cap genes for rAAV replication. After culture, HeLaRC cells were seeded in 24-well culture plates (Corning Inc., NY) at 5 × 10^4^ cells/well. Cells were infected with the rAAV vectors at MOIs 2 × 10^4^, 5 × 10^4^, 1 × 10^5^, 2 × 10^5^, and 5 × 10^5^, in triplicate. At 48 h post-infection, cells were washed with D-PBS (FUJIFILM Wako Pure Chemical Corp) and detached using TrypLE Select Enzyme (1X), with no phenol red (Thermo Fisher Scientific). Trypsin digestion was terminated by adding complete medium. Measurement of EGFP expression was performed with CytoFlex II Flow Cytometer (Beckman Coulter) using a fluorescein isothiocyanate (excitation: 498 nm, emission: 522 nm) channel with a threshold of 10,000 being used to identify EGFP-positive cells.

### BLI

BLI measurements were performed on the Octet HTX system (Sartorius). Octet ProA Biosensors (Sartorius) for BLI were hydrated by immersion in the AEX equilibration buffer for at least 30 min prior to use. For AAV9 and stress experiment to P2 of AAV8, we used formulation buffer instead of the AEX equilibration buffer. Samples were diluted with the AEX equilibration buffer or formulation buffer to a final concentration of 0.8–1.4 × 10^13^ vg/mL. A1 antibodies with 50 μg/mL were used because they specifically bind to the VP1u region. As a positive control for AAV8, full capsids were heated at 60°C for 15 min and subsequently re-fractionated by DGUC. Only the full-capsid fraction was then collected and used for analysis. As a positive control for AAV9 and stress experiment to P2 of AAV8, each P2 particle fractionated with AEX was heated at 60°C for 15 min. All measurements were performed at 30°C shaking at 1,000 rpm. Octet 384-well tilted-bottom microplate (Sartorius) was used for all BLI measurements. The measurements under A1 antibody immobilized condition consisted of the following five steps: (1) measure baseline using AEX equilibration buffer or formulation buffer for 100 s, (2) load 50 μg/mL A1 antibody onto the biosensors for 360 s, (3) measure baseline using AEX equilibration buffer or formulation buffer for 100 s, (4) associate AAVs with A1 antibodies for 800 s, and (5) dissociate AAVs from A1 antibodies for 400 s. The measurements under non-A1 antibody immobilized condition to investigate non-specific binding of AAVs to the biosensor consisted of the following three steps: (1) measure baseline using AEX equilibration buffer or formulation buffer for 100 s, (2) associate AAVs with the biosensors for 800 s, and (3) dissociate AAVs from the biosensor for 400 s. Octet Data Analysis software was used for data analysis.

### Forced stress experiment

Sample was subjected to buffer exchange with 200 mM NaCl and 0.001% P188 in Milli-Q water and then 2-fold diluted in the target pH buffer. Citrate and Tris salts were purchased from Nacalai Tesque (Kyoto, Japan), NaCl and HCl were from FUJIFILM Wako Pure Chemical (Osaka, Japan), and P188 was gifted by BASF (Ludwigshafen, Germany). Citrate salts were used at pH 2.5, and Tris was used at pH 9.5. The target pH buffers were prepared on the basis of calculations made using Buffer Maker software (v.1.1.0.0., ChemBuddy, Warsaw, Poland) to a 50 mM buffer concentration (300 mM ionic strength, 0.001% P188) and then filtered through a 0.22-μm filter.

rAAV in 200 mM NaCl 0.001% P188 was diluted 1:1 with the target Tris pH 9.5 buffer to perform alkaline treatment. Then, samples were aliquoted into 1.5-mL Safe-Lock tubes (Eppendorf, Hamburg, Germany) and stored at 25°C for 0, 3, and 7 days. Each was neutralized by 8:1 dilution with citrate pH 2.5 buffer after storage under these conditions, snap frozen in liquid nitrogen, and transferred to −80°C. Before use, they were thawed at room temperature.

rAAV in 200 mM NaCl 0.001% P188 was diluted 1:1 with the target citrate pH 2.5 buffer to perform acidic treatment. Then the samples were aliquoted into 1.5-mL Safe-Lock tubes (Eppendorf, Hamburg, Germany) and stored at 25°C for 0, 12.5, 60 min. Each was neutralized by 1:2 dilution with Tris pH 9.5 buffer after storage under these conditions, snap frozen in liquid nitrogen, and transferred to −80°C. Before use, they were thawed at room temperature.

### Analytical AEX

The analytical AEX was performed using a Thermo Scientific Vanquish UHPLC system coupled to a variable wavelength detector with ultraviolet absorbance at 280 nm and 260 nm and a fluorescence detector (excitation wavelength, 280 nm; emission wavelength, 350 nm). A strong anion exchange (quaternary ammonium) monolith (CIMac AAV e/f-0.1 (1.3), BIA Separation, Slovenia) was used for the separation at room temperature. Sample vials were placed at 4°C in the autosampler, and each injection contained 2.0 × 10^10^ viral particles for intact sample without stress treatment. Thermo Scientific Chromeleon 7 software was used for data processing and analysis.

The mobile phases used were as follows: mobile phase A—20 mM Tris solution (Nippon Gene), 1% (w/v) sucrose (Wako), 10 mM magnesium chloride (Nippon Gene), and 0.001% P188, pH 9.0; mobile phase B—1 M sodium chloride (Wako), 20 mM Tris solution (Nippon Gene), 1% (w/v) sucrose (Wako), 10 mM magnesium chloride (Nippon Gene), and 0.001% P188, pH 9.0. The flow rate was set at 1.0 mL/min, and the mobile phase gradient was 100% A for 1 min, then 0%–25% B from 1–10 min, 25%–100% B from 10–12 min, and 100%.

### Statistical analyses

Data are presented as the mean ± SD from three independent experiments. Statistical analyses were performed using an unpaired, two-tailed Welch’s *t* test for pairwise comparisons. For transduction assays, dose-response data were fitted using a four-parameter sigmoidal regression model, and differences between fitted curves were evaluated using an *F*-test. *p* < 0.05 was considered statistically significant.

## Data and code availability

The data from this study are available from the corresponding author upon reasonable request.

## Acknowledgments

This study was supported by Grants-in-Aid from “Research and Development of Core Technologies for Gene and Cell Therapy” supported by the 10.13039/100009619Japan Agency for Medical Research and Development (AMED) (grant numbers JP18ae0201002 and JP24se0123004h0101). We thank BASF for providing poloxamer 188. We also thank Saki Shimojo (10.13039/100015062The University of Osaka) for sample preparation.

## Author contributions

Conceptualization, Y.T. and S.U.; sample preparation, Y.T., H.M., X.L., and S.S.; methodology, Y.T., H.M., Z.Y., M.F., R.S., Y.Y., and S.U.; investigation and data analyses, H.M, H.K., Z.Y., R.S., and Y.Y.; writing – original draft, Y.T. and H.M.; writing – review and editing, Y.T., H.M., M.F., R.S., Y.Y., and S.U.; supervision, S.U.

## Declaration of interests

M.F. has relationship with U-Medico Inc. that includes employment and S.U. has a relationship with U-Medico Inc. as founder and CSO.

## Supplemental Information

Document S1. Figures S1–S3

Document S2. Article plus supplemental information
