# Supplementary material for: Anion-exchange chromatography separates structurally heterogeneous and low-potency particles in adeno-associated virus manufacture
Source: Mol Ther Adv. 2026 Jul 10;34(3):201812. doi: 10.1016/j.omta.2026.201812 (PMC13392945; doi:10.1016/j.omta.2026.201812)
Supplement: Document S1. Figures S1–S3 [file mmc1.pdf]

OMTA, Volume *34*

Supplemental information

Anion-exchange chromatography separates structurally heterogeneous and low-potency particles in adeno-associated virus manufacture

Yasuo Tsunaka, Haruka Makihira, Hanano Kono, Zhuolun Yang, Xiaofang Lyu, Sereirath Soth, Mitsuko Fukuhara, Risa Shibuya, Yuki Yamaguchi, and Susumu Uchiyama

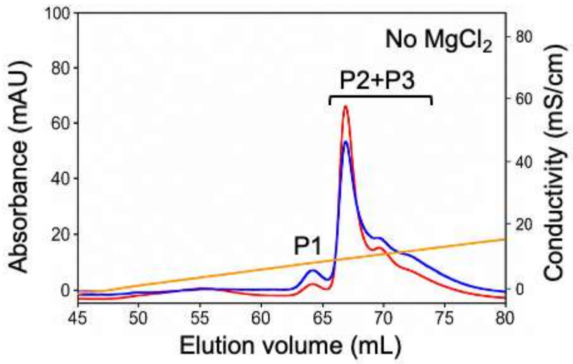

Figure S1. Chromatograms at 280 and 260 nm for anion-exchange chromatography (AEX) purification obtained using a linear sodium chloride gradient in the absence of $MgCl_2$. The A280 trace is shown as a blue line, the A260 trace as a red line, and the yellow line indicates conductivity.

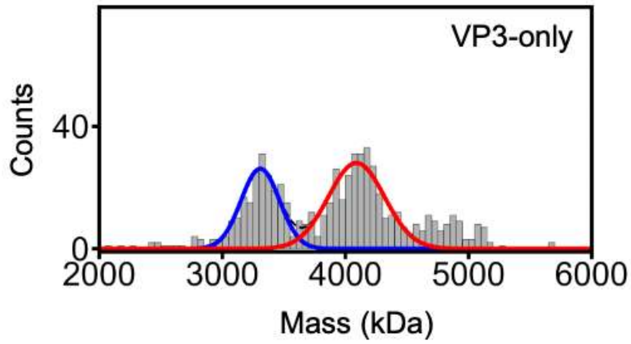

Figure S2. Representative mass photometry histograms of VP3-only, which is an AAV mutant with a capsid consisting entirely of VP3. The blue and red lines represent the areas of empty particles and full particles, respectively, based on their molecular mass.

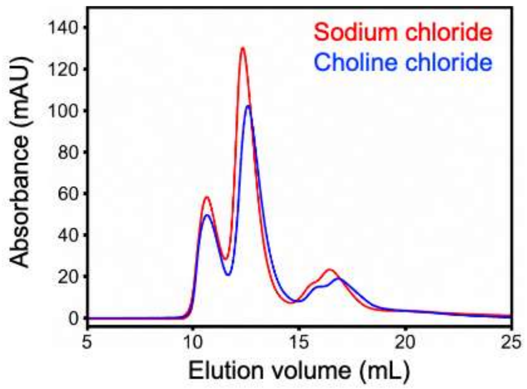

Figure S3. Comparison of AEX chromatograms of AAV8 monitored at 260 nm using linear sodium chloride (red) and choline chloride (blue) gradients.
